# Evaluation of the enhancement of photosynthetic rate in a komatsuna (*Brassica rapa* L. var. *perviridis*) canopy with upward lighting using an optical simulation in a plant factory with artificial light

**DOI:** 10.3389/fpls.2023.1111338

**Published:** 2023-03-24

**Authors:** Kota Saito, Eiji Goto

**Affiliations:** ^1^ Graduate School of Horticulture, Chiba University, Matsudo, Chiba, Japan; ^2^ Plant Molecular Science Center, Chiba University, Chiba, Japan

**Keywords:** abaxial surface, light distribution, light response curve of photosynthesis, vertical farming, photosynthetic photon flux density, plant three-dimensional model, light-emitting diode

## Abstract

In a plant factory with artificial light (PFAL), upward lighting is expected to prevent senescence and decrease in the photosynthetic capacity of the lower leaves in the canopy. Upward lighting may also increase the photosynthetic rate of a canopy by improving its photosynthetic photon flux density (PPFD) distribution. However, the net photosynthetic rate (Pn) of leaves is lower when the abaxial surface is irradiated than that when the adaxial surface is irradiated. The aim of this study was to estimate the PPFD in a PFAL and the Pn of plants using three-dimensional plant models and optical simulation. First, we measured the Pn of komatsuna (*Brassica rapa* L. var. *perviridis*) leaves under different conditions of the proportion (*p_ad_*) of PPFD on the adaxial surface to total PPFD on both surfaces and developed an equation for the light response curve of photosynthesis considering *p_ad_*. When PPFD was low, except when it was 30 and 70 µmol m^−2^ s^−1^, Pn increased as *p_ad_* increased, because the absorptance also increased with *p_ad_*. Under high PPFD conditions, Pn was maximized at 67–83% of *p_ad_* because the light would be distributed more efficiently for photosynthesis. Next, using optical simulation and the developed equation, we estimated the photosynthetic rate of a komatsuna canopy (CPn) under downward and upward lighting. The CPn increased by 1.08–1.13 times by combining downward and upward lighting due to the increase in the photosynthetic photon flux (PPF) of light incident on the canopy and the decrease in the spatial variation of PPFD on the leaves in the canopy. As the depreciation of lamps for upward lighting accounts for 7.5–9.0% of the production cost in a PFAL, even if the depreciation of lamps for upward lighting increased, enhancement of CPn by upward lighting would be cost-effective. We performed optical simulations under 220 conditions and evaluated them using CPn as an index. Moreover, we provided the proportion of PPF of upward lighting that improved CPn and discussed the reason for this improvement. The result shows that optical simulation is useful for evaluating the lighting design in a PFAL and analyzing the effects of the lighting design on the light environment and photosynthesis.

## 1 Introduction

Plant factories with artificial light (PFALs) and vertical farms are receiving increasing attention owing to unstable food supplies due to global population growth and climate change, as well as the expectation of high resource-use efficiency and low environmental burden compared to field and greenhouse agriculture (Kozai, 2013; Graamans et al., 2018; Kalantari et al., 2018; Kozai, 2019; Santiteerakul et al., 2020). In a PFAL, environmental factors such as light, temperature, humidity, and CO_2_ concentration can be controlled without being affected by the external environment, thus allowing us to create a favorable environment for plant growth (Kozai et al., 2006; Goto, 2012; Kalantari et al., 2018; Kozai, 2019). Therefore, stable and rapid plant production as well as better and more uniform product quality can be achieved.

Light is one of the most important environmental factors because it affects photosynthesis and morphogenesis (Kozai and Zhang, 2016). In PFAL, fluorescent lamps and light-emitting diode (LED) lamps are used as light sources. At present, LED lamps are primarily used because of their high controllability of light intensity and light quality and low power consumption. Lamps require electric energy, and the cost of their electric energy accounts for less than 20% of the production costs and 18% of the annual costs (Kozai, 2019; Kozai and Niu, 2020).

Numerous studies have investigated suitable light environments for plant growth and energy-efficient plant production using LED lamps. For example, the growth and energy efficiency of leafy lettuce (Kim et al., 2006; Shimizu et al., 2011; Zhang et al., 2018; Pennisi et al., 2020), basil (Dou et al., 2018; Hosseini et al., 2019; Pennisi et al., 2019; Pennisi et al., 2020), spinach (Zou et al., 2020), and strawberries (Warner et al., 2021) has been studied. Therefore, suitable light environment in a PFAL can improve the growth rate, quality of products, and energy use efficiency during production.

In a PFAL, a high planting density is preferable for increasing the yield per cultivation area and improving light-use efficiency (Yokoi et al., 2008; Kozai and Niu, 2016; Nicole et al., 2016). However, if the planting density is too high, the amount of light received by each plant will be reduced. This can result in undesirable morphologies, such as spindle growth and senescence of the lower leaves in a plant canopy. Furthermore, the photosynthetic capacity of the leaves will be reduced due to low light intensity (Yokoi et al., 2008; Brouwer et al., 2012; Joshi et al., 2017). Therefore, the yield rates decrease, and the burden of pruning increases (Zhang et al., 2015). Here, upward lighting from the bottom of the canopy is expected to prevent the senescence of the lower leaves and the decrease in photosynthetic capacity (Zhang et al., 2015; Joshi et al., 2017; Saengtharatip et al., 2021; Yamori et al., 2021).

The lower leaves receive less light as the leaves in the upper layers of the canopy absorb most of the light radiated on the canopy (Sassenrathcole, 1995; Li et al., 2014). If the photosynthetic characteristics of the leaves in the canopy are uniform, the net photosynthetic rate of the canopy (CPn) is maximized when the vertical distribution of the photosynthetic photon flux densities (PPFDs) in the canopy is uniform. In that case, upward lighting for the uniform vertical distribution of PPFDs in the canopy would increase CPn, even when the photosynthetic photon flux (PPF) emitted from the lamps is the same.

However, when only the abaxial surface of a leaf is irradiated, the net photosynthetic rate (Pn) can be lower than that when only the adaxial surface of a leaf is irradiated, even if the PPFDs on the adaxial and abaxial surfaces are the same. Such phenomena have been reported for soybean (Terashima, 1986), olive (Proietti and Palliotti, 1997), spinach (Sun and Nishio, 2001), European grapes (Palliotti and Cartechini, 2001), rose (Paradiso and Marcelis, 2012), and tobacco (Wang et al., 2020). Furthermore, Pn can be higher when both surfaces of a leaf are simultaneously irradiated compared to when only one surface of a leaf is irradiated, even if the total PPFDs are the same (Proietti and Palliotti, 1997; Palliotti and Cartechini, 2001; Sun and Nishio, 2001). If leaf Pn is varied with the proportion of PPFDs on adaxial and abaxial surfaces, uniformizing the vertical distribution of PPFDs in the canopy by upward lighting may not maximize CPn. Moreover, the effect of upward lighting on the canopy on CPn would vary with the leaf area index (LAI) of the canopy, the PPF of light from whole lamps, and the proportion of the PPFs of the downward and upward lighting.

Through optical simulations, the light environment in a PFAL (Akiyama and Kozai, 2016; Kim et al., 2020; Saito et al., 2020), the Pn of plants (Kim et al., 2020), and the bioactive compound contents of plants (Yoon et al., 2021; Yoon et al., 2022) have been estimated. Optical simulation can be performed by changing the positions, directions, and the spatial light distribution of the lamps. Therefore, the evaluation of various lighting designs using optical simulations is easier and faster than those with manual measurements.

In this study, we aimed to estimate the PPFD in a PFAL and the Pn of plants using three-dimensional plant models and optical simulation. To achieve this, we measured the Pn of komatsuna (*Brassica rapa* L. var. *perviridis*) leaves under different conditions of total PPFD and the proportion (*p_ad_*) of PPFD on the adaxial surface of the leaf to the total PPFD on both surfaces. We then developed a model equation for the light response curve (LRC) of photosynthesis that considers the *p_ad_*. Next, we performed optical simulations under different conditions of LAI and the PPFs of lamps for downward and upward lighting and estimated the PPFDs on the leaves of a canopy. Subsequently, we evaluated the effect of upward lighting on CPn under various lighting conditions. We also revealed that the Pn of leaves varied with the *p_ad_* and that upward lighting could enhance CPn.

## 2 Materials and methods

### 2.1 Plant material

Komatsuna (*Brassica rapa* L. var. *perviridis* ‘F1 Kuroba Pino Green’, Nakahara Seed Product Co. Ltd., Fukuoka, Japan) plant material was used. The cultivation was conducted twice, with 140 komatsuna plants per cultivation and the planting density was 250 m^−2^.The komatsuna plants were grown in a cultivation room in a closed plant production system at Chiba University. Komatsuna seeds were sown on a paper towel moistened with tap water. Germinated seedlings were placed on a polyurethane mat on the first day after sowing (DAS). Seedlings were irrigated with tap water until 6 DAS. On 7 DAS, komatsuna seedlings were transplanted onto cultivation panels. White LED lamps (XLX460NENT LE9, Panasonic Corporation, Osaka, Japan) with a color temperature of 5000 K were used as the light sources. The spectral distribution of the white LED lamps measured using a spectroradiometer (USR-45, Ushio Inc., Tokyo, Japan) is shown in **Figure S1**.

The nutrient solution was a 50% dilution of OAT house A recipe (OAT Agrio Co., Ltd., Tokyo, Japan). The nutrient solution contained 43.2, 26.4, 168.2, 80.2, and 18.2 mg L^−1^ of N, P, K Ca, and Mg, respectively, as well as 2.5 mg L^−1^ sodium thiosulfate. The electrical conductivity of the nutrient solution was 150–167 mS m^−1^. The pH was adjusted to 5.5–6.5 using a pH adjuster (OAT Agrio Co., Ltd., Tokyo, Japan). The nutrient solution was aerated during cultivation. PPFD, air temperature, relative humidity, and CO_2_ concentration were controlled during cultivation. The predetermined values for the environmental parameters during cultivation are shown in **Table S2**.

Uniformly grown plants were selected from 140 komatsuna plants and used for further experiments. The second and third leaves from the bottom of komatsuna plants on 16 and 18 DAS were used to measure leaf Pn. The second and third leaves from the bottom of plants on 16 DAS and the cotyledons and the first, second, third, fourth, and fifth leaves from the bottom of plants on 18 DAS were used to measure the leaf electron transport rate (ETR). In addition, 18 DAS plants were used to create three-dimensional (3D) models of plants and for leaf area measurements. The light environments in the 18 DAS canopy were estimated using optical simulations.

### 2.2 Measurement of the leaf net photosynthetic rate

#### 2.2.1 Net photosynthetic rate measurement device

**Figure 1:**
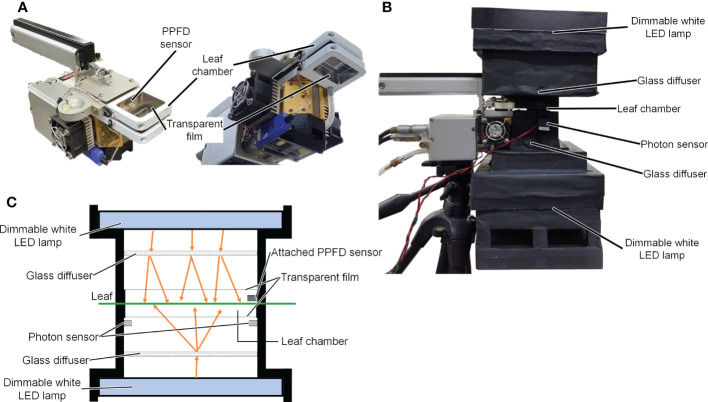
Images of LI-6400XT (LI-COR Inc.) with the transparent chamber **(A)** and the net photosynthetic rate measurement device **(B)**. Schematic diagram of the net photosynthetic rate measurement device **(C)**.

A LI-6400XT device (LI-COR Inc., NE, USA) with a transparent chamber top (adaxial side) and a transparent chamber bottom (abaxial side; LI-6400-08, LI-COR Inc. Inc., NE, USA) was used to measure the Pn of the leaves. Photographs of the Pn measurement device are shown in **Figures 1A, B**, and a schematic diagram is shown in **Figure 1C**. Two dimmable panel-type white LED lamps with a color temperature of 5500 K (ISLM-150X150-WW, CCS Inc., Kyoto, Japan) were used as light sources for the adaxial and abaxial sides of the Pn measurement device. The spectral photon distributions of the panel-type white LED lamps are shown in **Figure S2**. Glass diffusers (50 × 50 mm UV Fused Silica Ground Glass Optical Diffuser, Edmund Optics Japan, Tokyo, Japan) were used to diffuse light from the white LED lamps. Two-photon sensors (S1787-08, HAMAMATSU PHOTONICS K.K., Shizuoka, Japan) were placed on the abaxial side of the Pn measurement device to measure the PPFD on the abaxial side of a leaf surface. The relationships between the output voltages of the photon sensors and PPFDs were not linear at high PPFD. Therefore, one of the two photon sensors was shaded to allow measurement of a high PPFD (> 700 µmol m^−2^ s^−1^). The other sensor was used to measure low PPFD (< 700 µmol m^−2^ s^−1^). The relationships between the output voltages of the photon sensors and the PPFDs were determined using a PPFD sensor (LI-190SA, LI-COR Inc. Inc., NE, USA). A PPFD sensor mounted on the adaxial side of the Pn measurement device was used to measure the PPFD on the adaxial side of the leaf surface.

#### 2.2.2 Measurement conditions

During the Pn measurement, air temperature, relative humidity, CO_2_ concentration, and wind velocity in the leaf chamber were 25°C, 67–73%, 1000 µmol mol^−1^, and 0.34 m s^−1^, respectively.

The PPFD on a leaf was defined as follows:


(1) $$E=E_{ad}+E_{ab}$$

(2) $$p_{ad}=100\frac{E_{ad}}{E}$$

where *E* (µmol m^−2^ s^−1^) was the total PPFD of light incident on both the adaxial and abaxial surfaces of the leaf; *E_ad_* and *E_ab_* (µmol m^−2^ s^−1^) were the PPFDs of light incident on the adaxial and abaxial surfaces of the leaf, respectively; and *p_ad_* (%) is the proportion of *E_ad_* to *E*.

Pns were measured at 0, 30, 70, 150, 240, 330, 470, 700, 1100, and 1900 µmol m^−2^ s^−1^ of *E*. In addition, for each *E* level, *p_ad_* values were set at 1, 17, 33, 50, 67, 83, and 100%. However, for the third leaf of 16 DAS, Pns were measured at 120 and 170 µmol m^−2^ s^−1^ of *E* as well. After changing *E* or *p_ad_* and once the Pn became stable, the infra-red gas analyzers were matched and then logging was started. The data were logged at 1 s intervals for 60 s, and the average value of the data was used as the measured Pn. A new plant was used for each condition of PPFD, DAS, and leaf position. In total 94 plants were used for Pn measurement.

### 2.3 A light response curve of photosynthesis of a leaf

#### 2.3.1 Calculation of parameters of a light response curve of photosynthesis from the measured photosynthetic rates

The LRC of photosynthesis can be approximated using a non-rectangular hyperbola (Ögren and Evans, 1993). The equation for the non-rectangular hyperbola is as follows:


(3) $$Pn(E)=Pg(E)+Rd=\frac{\varphi_{P}E+P_{\max}-\sqrt{{\left(\varphi_{P}E+P_{\max}\right)}^{2}-4\varphi_{P}P_{\max}\theta_{P}}}{2\theta_{P}}+Rd$$

where *Pn(E)* (μmol m^−2^ s^−1^) is the Pn of a leaf irradiated with *E* μmol m^−2^ s^−1^, *Pg(E)* (μmol m^−2^ s^−1^) is the gross photosynthetic rate (Pg) of a leaf irradiated with *E* μmol m^−2^ s^−1^, *φ_P_* is the initial slope of the LRC, *P_max_* (μmol m^−2^ s^−1^) is the maximum value of the LRC, *θ_P_* is the convexity of the LRC, and *Rd* (< 0) (μmol m^−2^ s^−1^) is the dark respiration rate.

**Figure 2:**
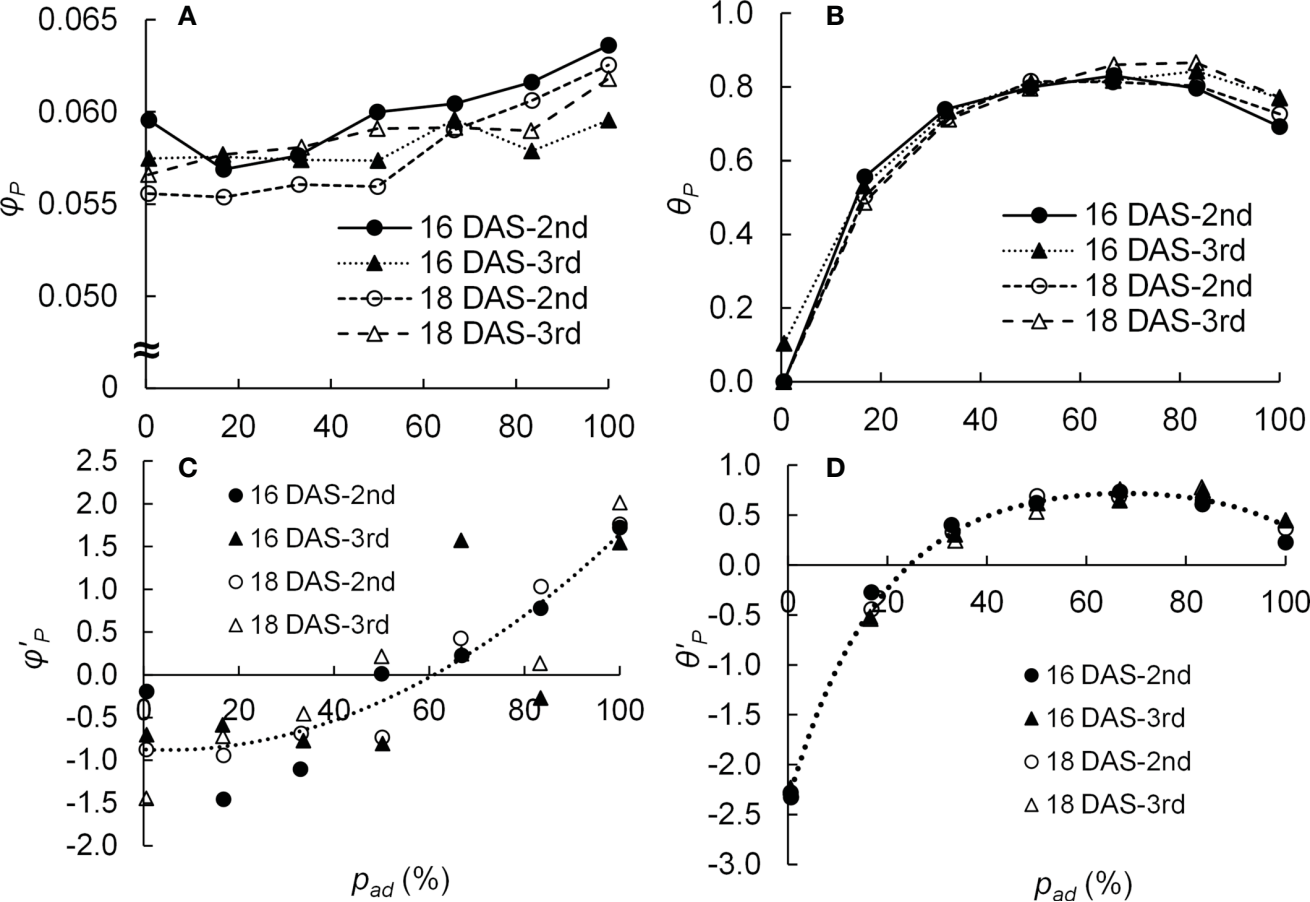
Relationship between the proportion (*p_ad_*) of photosynthetic photon flux density (PPFD) on the adaxial surface of a leaf to the total PPFD on both surfaces of a leaf and the initial slope (*φ_P_*) **(A)** as well as the convexity (*θ_P_*) **(B)** of the light response curve of photosynthesis of the 2nd and 3rd leaves of komatsuna sixteen and eighteen days after sowing (16 DAS and 18 DAS, respectively) (*n* = 21–26). The relationship between *p_ad_* and the z-score of the initial slope (*φ’_P_*) **(C)** as well as the z-score of the convexity (*θ’_P_*) **(D)** of the light response curve of photosynthesis of 2nd and 3rd leaves of komatsuna 16 DAS and 18 DAS (*n* = 21–26). *φ’_P_* and *θ’_P_* were calculated from *φ_P_* and *θ_P_* values at each DAS and leaf position (2nd or 3rd). The dotted lines in **(C, D)** were calculated using Eq. (4) and Eq. (5), respectively.

The parameters of LRC (*φ_P_*, *P_max_*, and *θ_P_*) were calculated from the measured Pn for each DAS (16 and 18 DAS), leaf position (second and third leaves), and *p_ad_* (1, 17, 33, 50, 67, 83, and 100%). The average Pn at 0 μmol m^−2^ s^−1^ of *E* was used as the *Rd* value. Some studies have shown the values of *P_max_* with irradiation to adaxial or abaxial surface were different (Wang et al., 2020). On the other hand, other studies have reported that when the photosynthesis of leaves was light-saturated, the Pns were similar regardless of whether the adaxial or abaxial surfaces were irradiated (Terashima, 1986; Palliotti and Cartechini, 2001). Therefore, the *P_max_* values were assumed to be the same for the same DAS and leaf position regardless of the *p_ad_* values. When fitting the curves using the least-squares method, the variance of the error was not uniform, and the curve was fitted using the generalized least-squares method. The generalized least-squares method was based on the method of Carroll and Ruppert (1982), and the weight function was the inverse of the square of *Pg(E)*. The generalized least-squares method was performed using the solver of Microsoft Excel (Microsoft Corporation, WA, USA). The obtained *φ_P_* and *θ_P_* values are shown in **Figures 2A, B**. The *P_max_* values were 53.7 and 52.5 μmol m^−2^ s^−1^ for the second and third leaves of 16 DAS and 50.2 and 53.7 μmol m^−2^ s^−1^ for the second and third leaves of 18 DAS, respectively.

#### 2.3.2 Calculation of the parameters of the light response curve of photosynthesis at arbitrary *p_ad_* values

The *φ_P_* and *θ_P_* obtained in Section 2.3.1 were standardized as *φ’_P_* and *θ’_P_* with respect to DAS and leaf position. The relationships between *p_ad_* and *φ’_P_* and between *p_ad_* and *θ’_P_* were assumed based on the relationships between *p_ad_* and *φ_P_* and between *p_ad_* and *θ_P_* (**Figures 2A, B**).


(4) $$\varphi {'}_{P}=a_{1}+a_{2}p_{ad}+a_{3}p_{ad}{}^{2}$$

(5) $$\theta {'}_{P}=b_{1}+\frac{b_{2}}{1+b_{3}e^{-b_{4}(p_{ad}-b_{5})}}-b_{6}e^{b_{4}(p_{ad}-b_{7})}$$

where *a_1_*, *a_2_*, *a_3_*, *b_1_*, *b_2_*, *b_3_*, *b_4_*, *b_5_*, *b_6_*, and *b_7_* are parameters of the equations. The parameters of the equations were obtained using the least-squares method. The obtained values of *a_1_*, *a_2_*, *a_3_*, *b_1_*, *b_2_*, *b_3_*, *b_4_*, *b_5_*, *b_6_*, and *b_7_* were −0.87, −0.026, 0.00028, −226, 267, 2.4, 0.0054, −98, 0.0017, and −5.7, respectively. The relationships between *p_ad_* and *φ’_P_* and between *p_ad_* and *θ’_P_* in Equations (4) and (5), respectively, are shown in **Figures 2C, D**.

Suppose that the increasing or decreasing trends of *φ_P_* and *θ_P_* with respect to *p_ad_* are equal to those of *φ’_P_* and *θ’_P_* with respect to *p_ad_*, regardless of DAS and leaf position. In this case, once the parameters of the LRC of photosynthesis at two *p_ad_* conditions (e.g., *p_ad_* = 0, 100%) can be obtained, the parameters of the LRC for arbitrary DAS and leaf position can be calculated. Then, the Pns for arbitrary *p_ad_* conditions can be calculated. Here, *φ_P_* was defined as follows:


(6) $$\varphi_{P}=c_{1}+c_{2}\varphi {'}_{P}$$

(7) $$c_{1}=\varphi_{P;ab}-\varphi {'}_{P;ab}\frac{\varphi_{P;ad}-\varphi_{P;ab}}{\varphi {'}_{P;ad}-\varphi {'}_{P;ab}}$$

(8) $$c_{2}=\frac{\varphi_{P;ad}-\varphi_{P;ab}}{\varphi {'}_{P;ad}-\varphi {'}_{P;ab}}$$

where *c_1_* and *c_2_* are the parameters determined for each DAS and the leaf position, respectively. *φ_P;ab_* and *φ’_P;ab_* are the values of *φ_P_* and *φ’_P_* when *p_ad_* is 0% and *φ_P;ad_* and *φ’_P;ad_* are the values of *φ_P_* and *φ’_P_* when the *p_ad_* is 100%. *θ_P_* values were obtained in the same manner.

**Figure 3:**
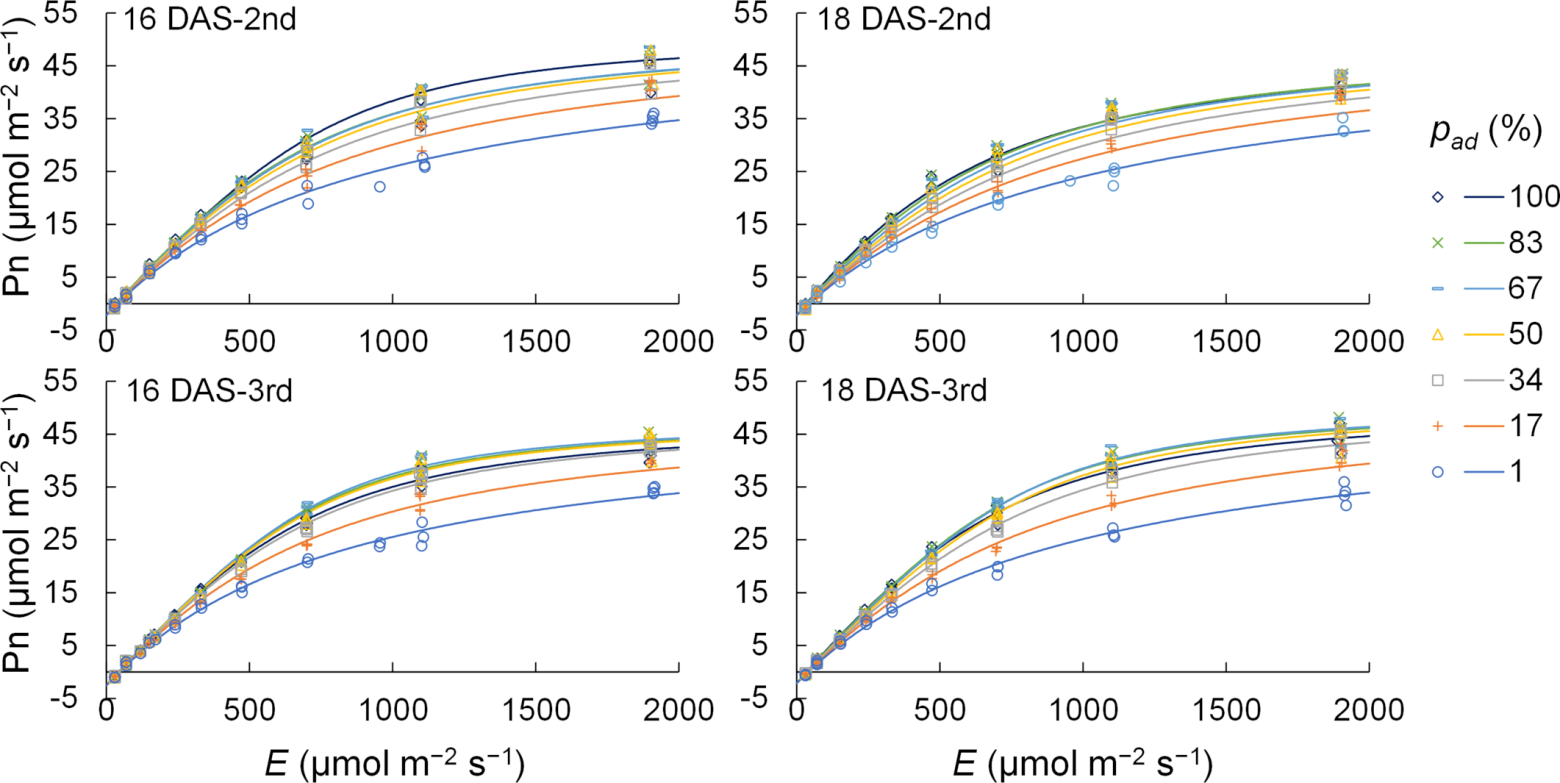
The measured (*n* = 148–178) and calculated values of net photosynthetic rate (Pn) of the 2nd and 3rd leaves of komatsuna 16 DAS and 18 DAS. *E* and *p_ad_* indicate the total photosynthetic photon flux density (PPFD) on the adaxial and abaxial surfaces of a leaf and the proportion of PPFD on the adaxial surface of a leaf to *E*, respectively. The light response curves of photosynthesis were obtained by the methods detailed in Section 2.3.2.

Five parameters, *φ_P;ad_*, *φ_P;ab_*, *P_max_*, *θ_P;ad_*, and *θ_P;ab_*, were determined for the second and third komatsuna leaves at 18 DAS using the generalized least squares method. *Rd* values were the average values of Pns under conditions of 0 μmol m^−2^ s^−1^ of *E*. Thus, LRCs considering *p_ad_* were obtained for the second and third komatsuna leaves at 18 DAS. The measured Pns and LRC values calculated using this method are shown in **Figure 3**. The accuracy of Pns calculated using this method is presented in **Table S3**.

#### 2.3.3 Calculation of the photosynthetic rates from electron transport rates

**Figure 4:**
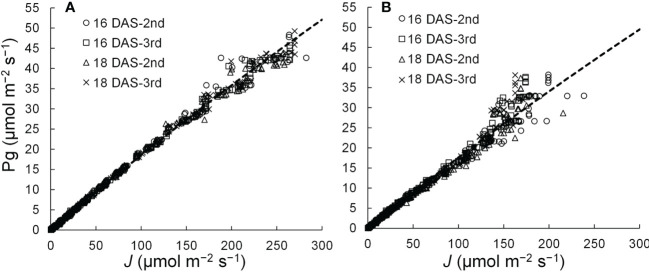
Relationships between the electron transport rates (*J*) and gross photosynthetic rates (Pg) of adaxial **(A)** and abaxial **(B)** surfaces of the 2nd and 3rd leaf of komatsuna 16 DAS and 18 DAS (*n* = 663–666).

A linear or curvilinear relationship exists between Pn and the ETR (Harbinson et al., 1990; Öquist and Chow, 1992; Yin et al., 2009). Therefore, the relationships between Pg and ETR at leaf positions where the Pns can be measured were determined, and the relationship was used to estimate the Pns from ETRs in those leaves. The chlorophyll fluorescence of komatsuna leaves was measured using a chlorophyll fluorescence measuring system (MINI-PAM; Heinz Walz GmbH, Baden-Württemberg, Germany). ETR (*J*; µmol m^−2^ s^−1^) was calculated using the equation described by Genty et al. (1989). The relationship between the ETR and Pg is shown in **Figure 4**. After Pn was calculated from the ETR by considering *Rd*, the LRC was obtained using the method described in Section 2.3.2. The *Rd* values were the average respiration rates of the second and third leaves at 16 and 18 DAS, respectively.

### 2.4 An optical simulation and estimation of photosynthetic rates

To estimate the CPn using optical simulation, it was necessary to create a cultivation system and lamps in optical simulation software (Radiance, Lawrence Berkeley National Laboratory, CA, USA; Ward, 1994), and to create 3D models of komatsuna plants using 3D modeling software (SketchUp 2017, Trimble Inc., CO, USA).

#### 2.4.1 Optical simulation software

A ray tracing software program, called Radiance (Berkeley Lab., CA, USA) was used to perform the optical simulation. The accuracy of the estimated PPFDs on a cultivation panel surface in a PFAL has been confirmed in our previous studies, where the mean absolute percentage error was found to be 4.50% (Saito et al., 2020). This was done by using this simulation software.

**Figure 5:**
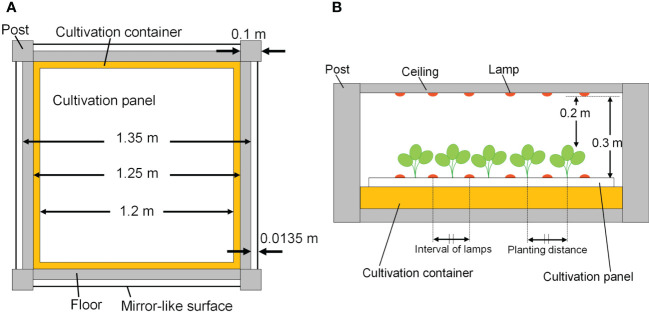
The top view of the cultivation system **(A)** and side elevation with lamps and plants **(B)**.

**Figure 6:**
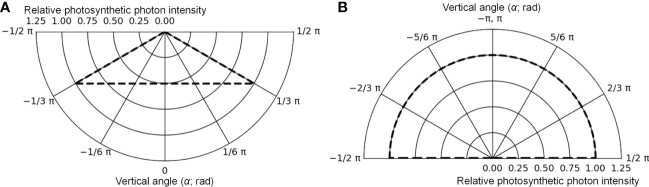
The photometric curves of lamps for downward lighting **(A)** and for upward lighting **(B)** in the optical simulation. The maximum values were converted to 1.


The cultivation system was created using Radiance functions, with the dimensions shown in **Figure 5** (125 cm D, 125 cm W, and 10 cm H), and was placed at the center of the floor. Virtual mirror-like surfaces, which were perfect specular reflectors, were placed 1.35 cm apart from the four sides of the floor, and it was surrounded by mirror-like surfaces. In this way, light coming from neighboring cultivation systems with the same design as a commercial PFAL could be reproduced. The lamps were 1.22 m in length and 0.03 m in width. The intervals between lamps were assumed to be equal to the planting distance. Lamps for upward lighting were assumed to be installed on the cultivation panel and placed at equal distances from neighboring plants (**Figure 5B**). Lamps for downward lighting were assumed to be placed exactly above lamps for upward lighting at a height of 0.3 m from the cultivation panel (**Figure 5B**). The lamps for downward lighting were placed approximately 0.2 m above the plant canopy. The spatial light distributions of the lamps were set to irradiate the canopy uniformly (**Figure 6**) and are given by the following equations:

(9) $$I_{dw}(\alpha )=I_{dw}(0)\frac{1}{\cos\alpha }\left(-\frac{\pi }{3}\leq \alpha \leq \frac{\pi }{3}\right)$$

(10) $$I_{uw}(\alpha )=I_{uw}(\pi )\left(-\pi \leq \alpha \leq -\frac{\pi }{2},\frac{\pi }{2}\leq \alpha \leq \pi \right)$$

where *I_dw_(α)* (μmol s^−1^ sr^−1^) was the photosynthetic photon intensity (PPI) at *α* (rad) of the vertical angle to the emitting surface of the lamp for downward (*dw*) lighting and *I_uw_(α)* (μmol s^−1^ sr^−1^) was the PPI at *α* (rad) of the vertical angle to the emitting surface of the lamp for upward (*uw*) lighting. For horizontal angles to the emitting surface of the lamp, *I_dw_(α)* and *I_uw_(α)* were assumed to remain consistent. *I_dw_(0)* and *I_uw_(π)* values were determined using the PPFs of the lamps. The spectral distributions of the lamps were assumed to correspond to those of the lamps used for cultivation (**Figure S1**). Because Radiance simulates three types of light, R, G, and B, the photon flux ratios of light at 400–500, 500–600, and 600–700 nm wavelengths were input into the channels of B, G, and R, respectively, based on the spectral distributions of the lamps.

The optical properties of the komatsuna plants and the structures of the cultivation system could be input into the Radiance simulation. The optical properties of an object can be input into each RGB channel. A reflection sheet (FEB#110; Yupo Corporation, Tokyo, Japan) was attached to the ceiling of the cultivation system. The spectral distributions of reflection and transmission of the leaves, reflection sheet, and cultivation panel were measured using a UV-Vis-NIR spectrophotometer (V-750; JASCO Corporation, Tokyo, Japan) equipped with an integrating sphere unit (ISV-922; JASCO Corporation, Tokyo, Japan). Because the reflectance of the container, the post of the cultivation system, and the petiole could not be measured, the reflectance in the Radiance simulation was obtained using Colour Picker, which could calculate the reflectance from the appearance of the color. The optical properties of the objects used in the simulations are presented in **Table 1**.

**Table 1:** The optical properties of items in the optical simulation.

Item	Reflectance (%)			Transmittance (%)		
	R	G	B	R	G	B
Leaf	3.31	5.65	1.90	4.33	10.4	0.637
Petiole	34.1	55.2	7.29	8.11	13.4	1.41
Cultivation panel	89.5	89.9	90.4	0	0	0
Cultivation container	73.4	35.4	0.500	0	0	0
Floor and Post	70.0	70.0	70.0	0	0	0
Ceiling	90.9	91.3	91.4	0	0	0

In this table, the mean values of the reflectance and transmittance of the leaves at each leaf position are shown. The leaf reflectance and transmittance values were input for each leaf position in the optical simulation.

#### 2.4.2 3D models of komatsuna plants

**Figure 7:**
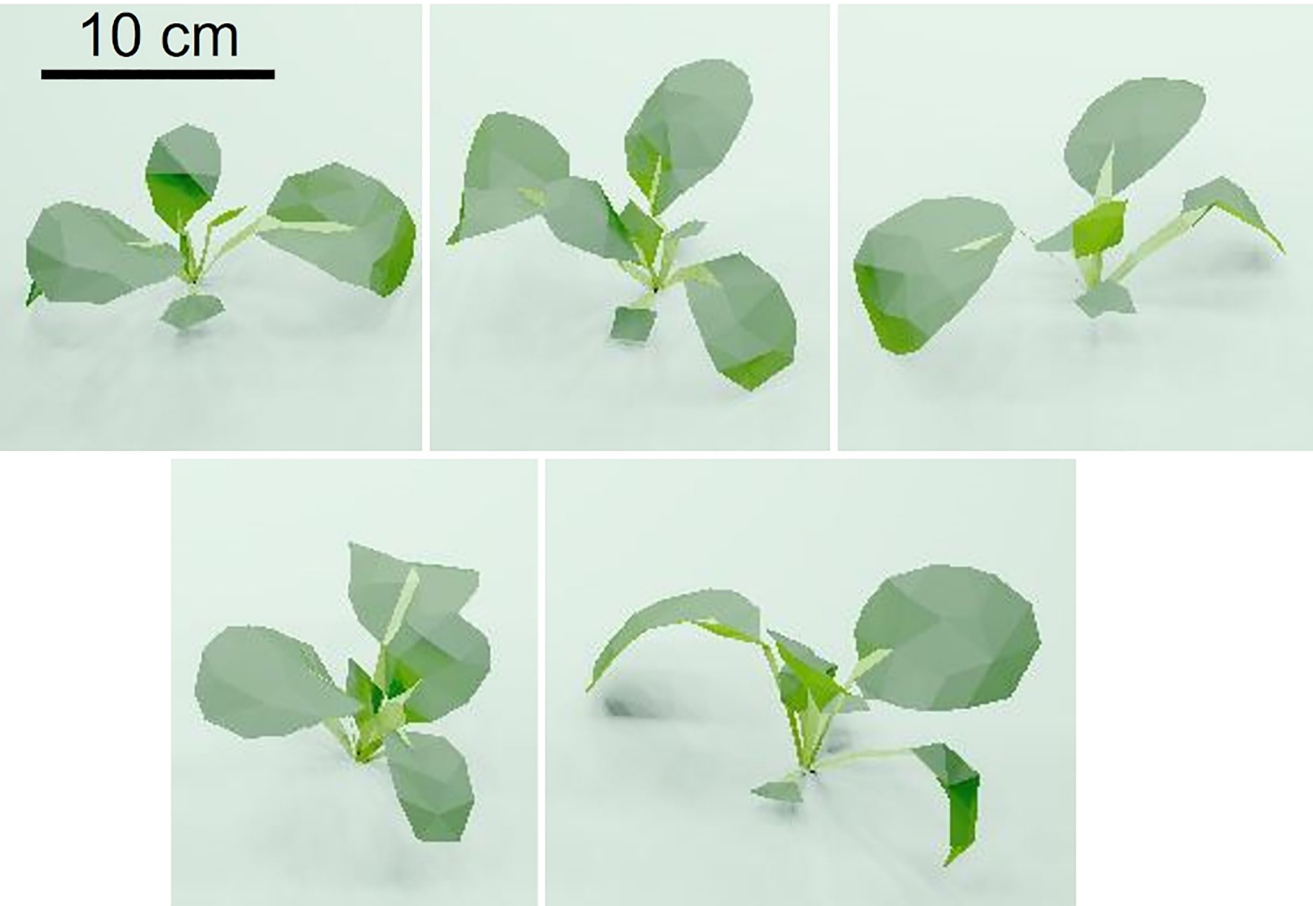
Images of five komatsuna three dimensional models used in the optical simulation by Radiance.

The 3D models of komatsuna plants at 18 DAS were created using the Structure from Motion method (**Figure 7**).

##### 2.4.2.1 Reconstruction of 3D models of leaf blades

The point cloud data obtained with Metashape (Agisoft, St. Petersburg, Russia) include the X, Y, and Z coordinates of the points, color information (RGB color model), and the X, Y, and Z components of the normal to the points. Videos were captured using a smartphone camera to obtain images of the plants. The videos had a pixel count of 1920 × 1080 and 30 frames. Four markers were placed around the plant during video capture and used to calibrate the distance scale of the point cloud. The videos were imported into Metashape, and approximately 1000 images were created. Using the images, first, the processed point cloud data of the plants were obtained.

Next, the points of the leaf blade were extracted by manual work and logistic regression analysis from the first processed point cloud data. The point cloud of the leaf blade was converted into a mesh model using Poisson Surface Reconstruction (Kazhdan et al., 2006), a function of CloudCompare (Électricité de France, 8th Arrondissement of Paris, France). In this process, the areas of the mesh model of individual plants were set to 90–110% of the leaf areas of individual plants measured using a leaf area meter (LI-3100, LI-COR Inc., NE, USA). The number of triangles for the leaf blade models of the individual plants was set to 100 to reduce the computation volume.

##### 2.4.2.2 Creation of 3D models of petioles

Petiole mesh models were created using SketchUp 2017. The leaf blade mesh models created in 2.4.2.1 were imported into SketchUp 2017. The 3D models of the leaf blades and petioles created as described above were converted into a usable file in Radiance (.rad) using su2rad, which is a function of SketchUp 2017. Five rad files were created as 3D models of five komatsuna plants (**Figure 7**). The komatsuna 3D models recorded in these files were placed in a cultivation system using xform, a function in Radiance.

#### 2.4.3 Calculation of estimated photosynthetic rates

In the optical simulation, the PPFDs of the centers of gravity of all triangles comprising the leaf blade models were estimated. For each triangle, the estimated PPFDs were input into Equations (1)–(3) to obtain Pn at the center of gravity of the triangles. The Pn per triangle can be obtained by multiplying Pn by the area of a triangle. This calculation was performed for all triangles comprising the all-leaf blade models, and the values were summed to obtain CPn.

#### 2.4.4 Conditions for optical simulations

Optical simulations were performed under conditions with different LAI and PPFs of the lamps for downward and upward lighting. The lamps’ total PPF for downward and upward lighting was defined as *Φ_lamps_* (μmol s^−1^), and the proportion of PPF of the lamps for downward lighting to *Φ_lamps_* was defined as *p_dw_* (%).

LAI was set at five levels (0.5, 1.5, 2.5, 3.5, and 4.5), *Φ_lamps_* was set at four levels (122, 244, 366, and 488 µmol s^−1^), and *p_dw_* was set at 11 levels (0, 10, 20, 30, 40, 50, 60, 70, 80, 90, and 100%). In total, 220 simulations were conducted. When *Φ_lamps_* was 122 µmol s^−1^, the estimated average PPFD on the leaves of the komatsuna canopy with 1.0 of LAI was approximately 100 µmol m^−2^ s^−1^. The planting distance was calculated to achieve the intended LAI because the average leaf area of the 18 DAS komatsuna plants was 110.2 cm^2^. The 3D models were placed on the cultivation system using xform, a function of Radiance, to set the intended planting distance. The PPF per lamp was calculated from *Φ_lamps_*, the number of lamps used in each LAI condition, and *p_dw_*.

## 3 Results

### 3.1 Net photosynthetic rates of a komatsuna leaf

**Figure 8:**
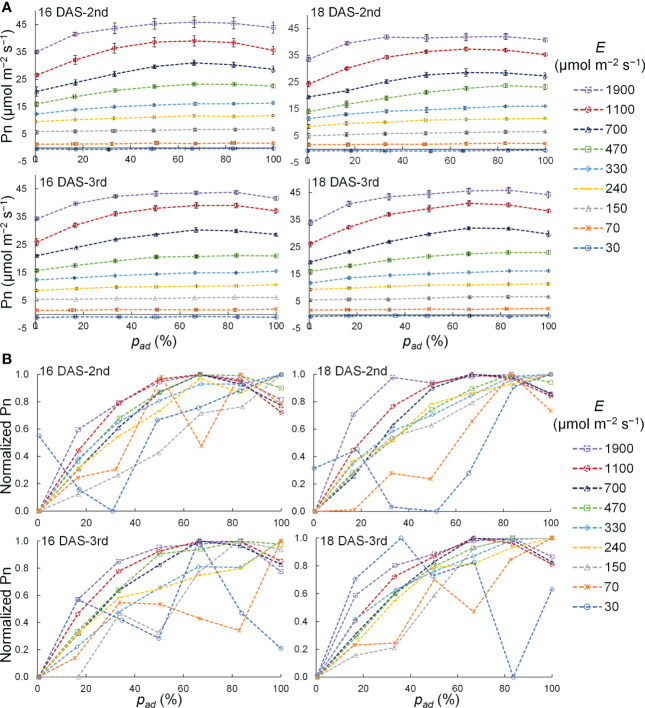
Relationship between the proportion (*p_ad_*) of the photosynthetic photon flux density (PPFD) on a leaf adaxial surface to the total PPFD on the adaxial and abaxial surfaces of a leaf (*E*) and net photosynthetic rate (Pn) **(A)** as well as normalized Pn **(B)** of the 2nd and 3rd leaves of komatsuna 16 DAS and 18 DAS with different *E.* In **(A)**, these plots represent the average values, and the horizontal and vertical lines represent standard error at each condition (*n* = 2–5). In **(B)**, the plots represent the normalized values that were calculated by normalizing the Pn values at each *E* condition.

The relationship between *p_ad_* and the Pn of a komatsuna leaf at 16 DAS and 18 DAS is shown in **Figure 8A**. The variation in Pn with *p_ad_* tended to increase as the PPFD increased for all DAS and leaf positions. To analyze the relationship between *p_ad_* and Pn in detail, normalized Pn values with respect to the PPFD level were calculated, as shown in **Figure 8B**. The Pn increased with the *p_ad_* when *E* was 150–330 μmol m^−2^ s^−1^, and the Pn tended to be the maximum at 67–83% of the *p_ad_* when *E* was 470 μmol m^−2^ s^−1^ or larger. Under the conditions with 30 and 70 μmol m^−2^ s^−1^ of *E*, no trend concerning the *p_ad_* was observed.

### 3.2 Estimated photosynthetic rates of a komatsuna canopy

The optical simulation took the reflections of light on the komatsuna leaves and the surfaces of the cultivation system, the transmission of light on the leaves, and the incidence of light on both the adaxial and abaxial surfaces of all leaves within the canopy, into account. These phenomena occur in an actual PFAL. Moreover, Pn of all leaves could be estimated considering *p_ad_* using an optical simulation.

**Figure 9:**
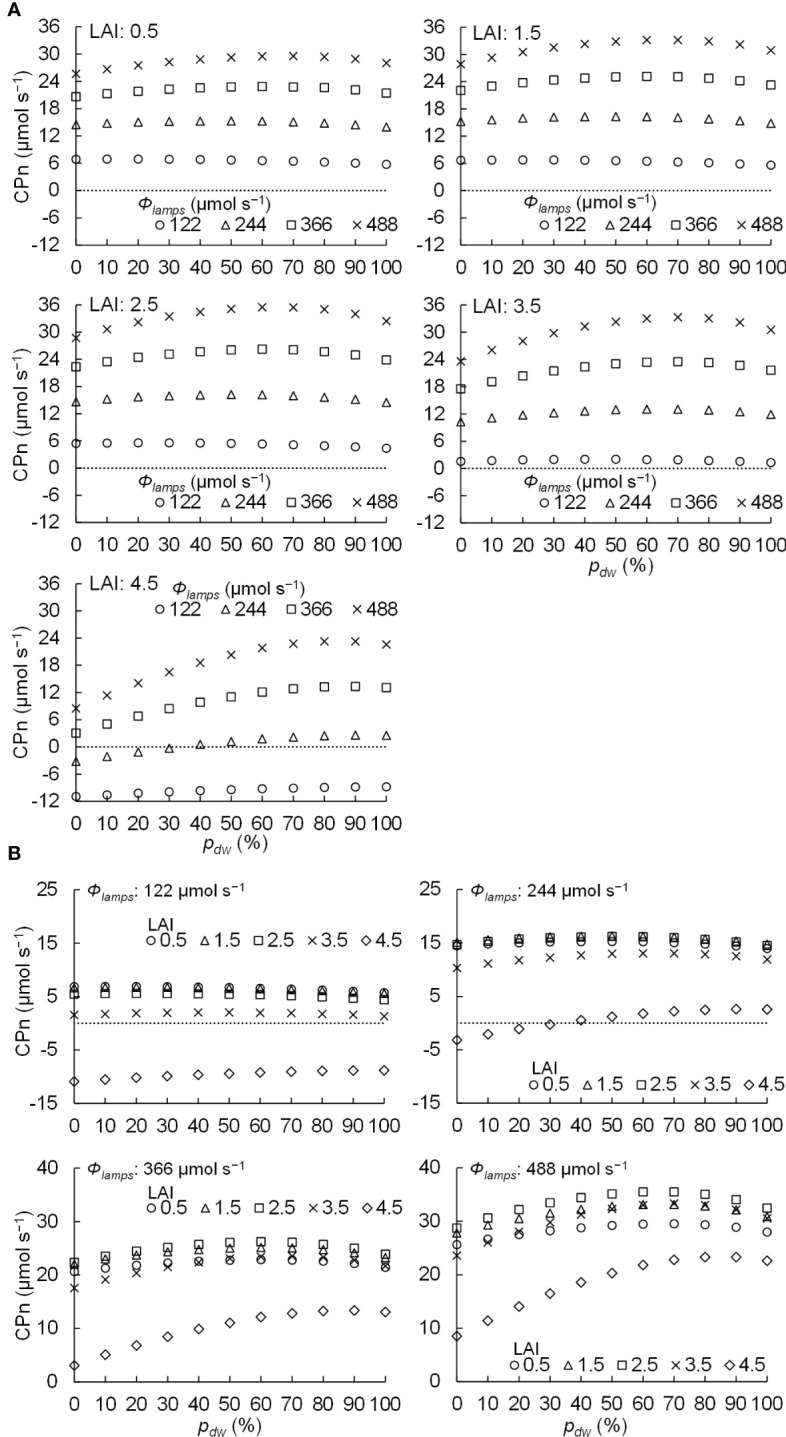
Relationship between the proportion (*p_dw_*) of the photosynthetic photon flux of lamps for downward lighting to that of whole lamps for downward and upward lighting (*Φ_lamps_*) and the net photosynthetic rates of the komatsuna canopy (CPn) in different conditions of leaf area index (LAI) **(A)** and *Φ_lamps_* **(B)**.

The relationships between *p_dw_* and CPn under different LAI and *Φ_lamps_* levels are shown in **Figure 9**. **Figure 9A** shows the relationship between *p_dw_* and CPn under different *Φ_lamps_* conditions for the same LAI, and **Figure 9B** shows the relationship between *p_dw_* and CPn under different LAI conditions for the same *Φ_lamps_*. When the LAI was 0.5–3.5, the CPn was positive in every condition. However, when the LAI was 4.5 and *Φ_lamps_* was 122 or 244 µmol s^−1^, some conditions resulted in negative CPn due to excess LAI.

**Figure 10:**
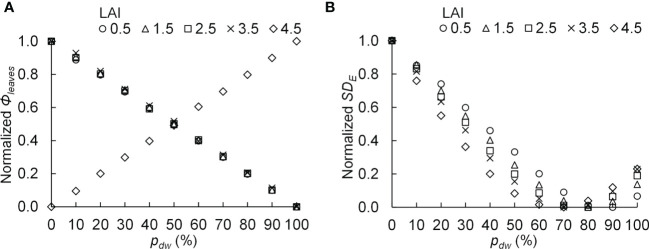
The relationship between the proportion (*p_dw_*) of the photosynthetic photon flux of lamps for downward lighting to that of whole lamps for downward and upward lighting and the normalized photosynthetic photon flux incident on the komatsuna canopy (normalized *Φ_leaves_*) **(A)**, as well as the normalized standard deviation of the photosynthetic photon flux density on komatsuna leaves within the canopy (normalized *SD_E_*) **(B)**. The average values of normalized values were calculated for each leaf area index (LAI) condition.

The PPF of light incident on the komatsuna canopy was defined as *Φ_leaves_*, and the standard deviation of the estimated PPFD on komatsuna leaves was defined as *SD_E_*. *SD_E_* is an index of variation in PPFD within a canopy. A smaller value indicates greater uniformity in the vertical distribution of PPFD within the canopy. If the photosynthetic characteristics of the leaves in the canopy are uniform and *Φ_leaves_* values are equal, the smaller the *SD_E_*, the larger the CPn. The relationships between *p_dw_* and the normalized *Φ_leaves_* with respect to each level of LAI and *Φ_lamps_*, as well as the normalized *SD_E_* with respect to each level of LAI and *Φ_lamps_*, are shown in **Figure 10**.

**Table 2** shows the *p_dw_* values that maximized the CPn at each level of LAI and *Φ_lamps_*. It also shows the ratios of the gross photosynthetic rates of the canopies in the conditions to those in the only downward lighting condition (*p_dw_* = 100 %). From **Table 2** and **Figure 9A**, at 0.5–2.5 of LAI and 122 µmol s^−1^ of *Φ_lamps_*, the CPn values were at its maximum when *p_dw_* was 20% and decreased as *p_dw_* increased. At 0.5–3.5 of LAI and 244, 366, and 488 µmol s^−1^ of *Φ_lamps_*, the CPn values were at its maximum when *p_dw_* was 50–70%, and decreased as *p_dw_* increased or decreased away from those values. Furthermore, the values of *p_dw_*, at which the CPn was at its maximum, was the same or greater under the conditions with 3.5 and 4.5 of LAI in comparison to 0.5–2.5 of LAI. As shown in **Figure 9B**, under any LAI condition, the higher the *Φ_lamps_*, the higher the variation in CPn, depending on the *p_dw_*.

**Table 2:** The proportion (*p_dw_*; %) of the photosynthetic photon flux of lamps for downward lighting to that of whole lamps for downward and upward lighting (*Φ_lamps_*) that maximize the net photosynthetic rates of the komatsuna canopy in each condition of the leaf area index (LAI) and *Φ_lamps_*.

		*Φ_lamps_* (µmol s^−1^)			
		122	244	366	488
LAI	0.5	20* (1.13)	50 (1.08)	60 (1.06)	70 (1.05)
	1.5	20 (1.12)	50* (1.08)	60 (1.07)	70 (1.07)
	2.5	20 (1.11)	50 (1.08)	60* (1.08)	60* (1.08)
	3.5	50 (1.06)	60 (1.05)	70 (1.06)	70 (1.07)
	4.5	100 (1.00)	90 (1.00)	90 (1.01)	80 (1.02)

The values with asterisks indicate the *p_dw_* values that maximize the net photosynthetic rates in each *Φ_lamps_* condition. The values in the brackets indicate the ratio of gross photosynthetic rates in the p_dw_ conditions to those in the condition of *p_dw_* = 100%.

At 0.5–3.5 of LAI, the *Φ_leaves_* became smaller as *p_dw_* increased, whereas under 4.5 of LAI, the *Φ_leaves_* increased as *p_dw_* increased (**Figure 10A**). The relationships between *p_dw_* and *SD_E_* were similar for all LAI conditions, and the values of *SD_E_* were the smallest under the conditions with 70–90% of *p_dw_* (**Figure 10B**).

## 4 Discussion

### 4.1 Relationship between *p_ad_* and photosynthetic rate of a komatsuna leaf

Pn varied with *p_ad_* when *E* was constant, except for conditions with 30 and 70 μmol m^−2^ s^−1^ of *E*. Previous studies have shown similar trends in Pn with irradiation on the adaxial, abaxial, and both surfaces of a leaf. For example, even if the PPFDs were the same, Pn was higher when only the adaxial surface of a leaf was irradiated than when only the abaxial surface of a leaf was irradiated (Terashima, 1986; Paradiso and Marcelis, 2012; Wang et al., 2020). Furthermore, Pn was higher when both the adaxial and abaxial leaf surfaces were irradiated simultaneously than when only the adaxial surface of a leaf was irradiated, even if the total PPFDs on both surfaces were the same (Palliotti and Cartechini, 2001; Sun and Nishio, 2001). When *E* was 30 and 70 μmol m^−2^ s^−1^, Pn was small; thus, accidental errors were large relative to the measured values. Therefore, no trends were observed in the Pn depending on *p_ad_*. When *E* was greater than 70 μmol m^−2^ s^−1^, Pn varied with *p_ad_*. This may be due to the differences in the optical properties of the adaxial and abaxial surfaces of the leaf, the degree of attenuation within the leaf of light incident from the adaxial and abaxial surfaces, and the gradients in the photosynthetic capacity of chloroplasts within the leaf. In the following section, we discuss the possible reasons for these differences in detail.

**Figure 11:**
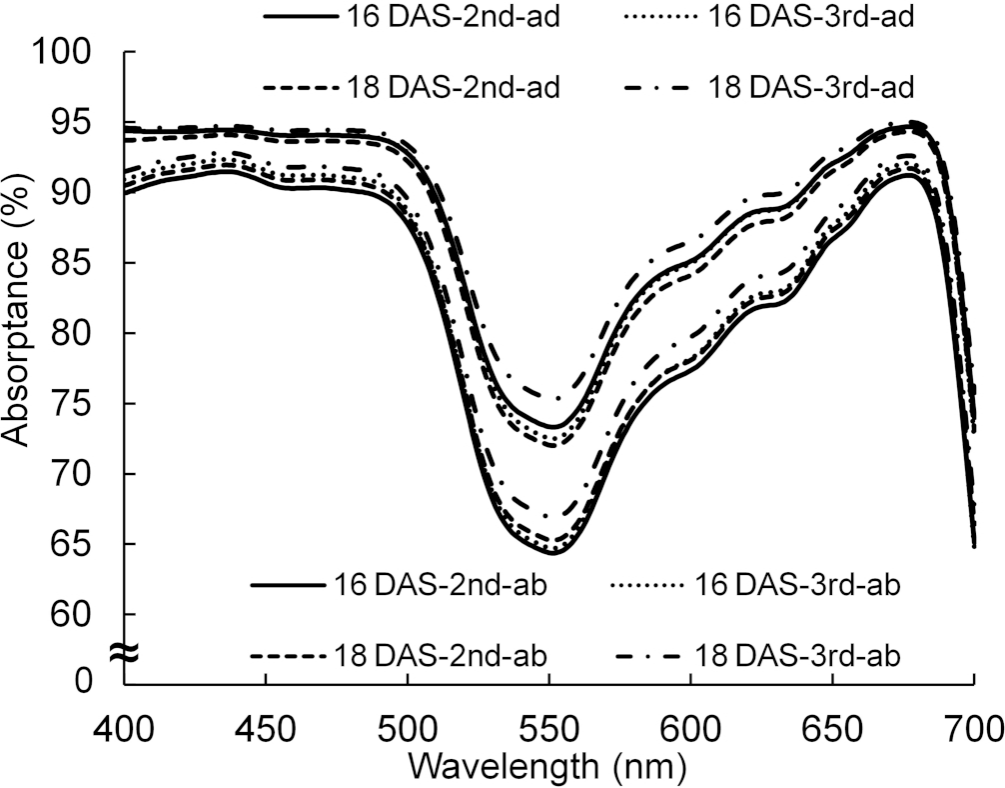
The spectral distributions of absorptance of adaxial (ad) and abaxial (ab) surfaces of the 2nd and 3rd leaves of komatsuna 16 DAS and 18 DAS (*n* = 10). The spectral distributions were calculated from the spectral distributions of reflectance and transmittance measured by a spectrophotometer (V-750, JASCO Corporation, Tokyo, Japan) with an integrating sphere (ISV-922, JASCO Corporation, Tokyo, Japan). The spectral distributions of absorptance of the komatsuna leaves in the other leaf positions in 18 DAS were similar to those of the 2nd and 3rd leaves of komatsuna 18 DAS.

By using the spectral distributions of photons from the lamps and the absorptance of a leaf in **Figures 3** and **11**, the absorptances, when the light was incident from the adaxial or abaxial surface of the leaf, were calculated every 1 nm. The average absorptance (400–700 nm) of the abaxial surface was approximately 93% of that of the adaxial surface.

Palisade tissue allows light to be transmitted more easily than spongy tissue. On the other hand, the structure of spongy tissue allows light to scatter and be absorbed more easily. Therefore, the incident light from the palisade tissue is less likely to be attenuated (Brodersen and Vogelmann, 2010; Timonin, 2017). Therefore, if mesophyll cells of the komatsuna leaf were differentiated into palisade and spongy tissue, the gradient of PPFD in the leaf from the adaxial to abaxial surfaces would be smaller when the light was incident from the adaxial surface compared to when the light was incident from the abaxial surface.

The komatsuna plants used in this study were grown under downward light. Therefore, the light was mainly incident on the adaxial surface of the leaves. In this case, the PPFD of the leaves was relatively high on the adaxial surface and relatively low on the abaxial surface. Chloroplasts within a leaf can adapt to the light environment in which the leaf develops (Terashima, 1986). In leaves exposed to light incident on the adaxial surface, the ribulose 1,5-bisphosphate carboxylase/oxygenase (Rubisco)/chlorophyll ratio, chlorophyll a/b ratio, Rubisco concentration, Rubisco activity, and CO_2_ fixation rate were higher on the adaxial side than on the abaxial side (Nishio et al., 1993; Terashima and Hikosaka, 1995; Sun and Nishio, 2001; Terashima et al., 2016). Rubisco content is correlated with the CO_2_ fixation rate (Nishio et al., 1993), and the chlorophyll a/b ratio reflects the ratio of the reaction center and light-harvesting pigment protein in the photosystem. Therefore, the photosynthetic activity of chloroplasts was higher on the adaxial side than on the abaxial side, and chloroplasts on the adaxial side were more adapted to high PPFD by downward lighting.

Thus, the effect of the *p_ad_* on the Pn of a leaf can be explained as follows. Under *E* conditions lower than 330 μmol m^−2^ s^−1^, the slope of the LRC of photosynthesis was the product of the quantum yield of photosynthesis and the light absorptance in the leaf (Terashima and Saeki, 1985), and Pn increased linearly with increasing PPFD on the leaf. Therefore, if the quantum yield of photosynthesis is approximately the same within a leaf, the higher the leaf absorptance, the higher the Pn. This indicates that under low PPFD conditions, the percentage of light incident from the adaxial surface with higher absorptance was higher; that is, the higher the *p_ad_*, the higher the Pn.

Under *E* conditions higher than 470 μmol m^−2^ s^−1^, if the photosynthetic activity of the chloroplasts is uniform, Pn reaches a maximum when the PPFD in the leaf is uniform. Furthermore, if the extinction coefficients of the palisade and spongy tissues are equal, Pn becomes maximum when both sides of the leaf are irradiated at an equal PPFD (*p_ad_* = 50). However, it is considered that the extinction coefficient of palisade tissue is lower than that of sponge tissue, the photosynthetic activity of chloroplasts is higher on the adaxial side than on the abaxial side, and chloroplasts on the adaxial side are more adapted to high PPFD (Nishio et al., 1993; Terashima and Hikosaka, 1995; Sun and Nishio, 2001; Terashima et al., 2016). Thus, it is considered that Pn was higher with a higher percentage of PPFD on the adaxial surface (*p_ad_* = 67–83%) than that with equal PPFD on both sides of the leaf (*p_ad_* = 50%). This is because light energy was distributed more efficiently to each chloroplast.

### 4.2 Relationships between *p_ad_* and parameters of a light response curve of photosynthesis

*φ_P_* is the initial slope of the LRC of photosynthesis and reflects Pn under low PPFD conditions, as described in Section 4.1. Under low PPFD conditions, the slope of the LRC of photosynthesis is the product of the quantum yield of photosynthesis and the absorptance of light in the leaf (Terashima and Saeki, 1985). Therefore, the absorptance of the leaf increases as the *p_ad_* increases, and consequently, *φ_P_* increases with *p_ad_*.

*θ_P_* is the convexity of the LRC of photosynthesis, which indicates the ease of light saturation of Pn. If *P_max_* is constant, the light-use efficiency of photosynthesis increases as *θ_P_* increases.

Light incident on the abaxial surface is scattered and absorbed by the spongy tissue, resulting in a steeper gradient of PPFD between the adaxial and abaxial surfaces compared to the light incident on the adaxial surface (Terashima and Saeki, 1985; Terashima and Hikosaka, 1995). In addition, more light incident on the abaxial surface is likely to be absorbed by chloroplasts, with relatively low photosynthetic activity on the abaxial side (Nishio et al., 1993; Sun and Nishio, 2001; Terashima et al., 2016). When light is incident only on the abaxial surface (*p_ad_* = 0), much of the light can be absorbed by the spongy tissue and only a small amount of light can reach the palisade tissue. Therefore, the increase in PPFD of the light reaching the palisade tissues is small relative to that of the light reaching the abaxial surface (*E_ab_*). Thus, it is difficult to saturate the Pn of the leaves. In particular, when a leaf is irradiated with light with a PPFD that causes the Pn of chloroplasts in the abaxial side to be light-saturated, the light energy absorbed by the chloroplasts would not be efficiently used for photosynthesis.

When the *p_ad_* was 0%, excessive light would be absorbed by the chloroplasts on the abaxial side, but when the *p_ad_* was 17–83%, some of the excessive light could be absorbed by the chloroplasts on the adaxial side. Thus, when the light was incident from the adaxial side, more light could be used for photosynthesis by the chloroplasts on the adaxial side, which might not be light-saturated compared to the condition with 0% of the *p_ad_*. Therefore, even if *E* is constant, the Pn would be higher at 17–83% of the *p_ad_* than at 0 of the *p_ad_*.

When light is incident on the adaxial surface only (*p_ad_* = 100%), the Pn of the leaf is difficult to be saturated compared to the conditions with 50, 67, and 83% of the *p_ad_* because the PPFD of the light reaching the abaxial surface is low, and the Pn of the chloroplasts on the abaxial side is difficult to be saturated, contrary to the condition with 0% of the *p_ad_*. However, palisade tissue allows light to be transmitted more easily than sponge tissue, and in a leaf developed with irradiation on the adaxial side, the photosynthetic activity of the chloroplasts might be higher on the adaxial side than on the abaxial side. Therefore, it is considered that since chloroplasts adapted to high PPFD are harder to be saturated, the Pn of the leaf was harder to be saturated at *p_ad_* = 100%, than at *p_ad_* = 0%.

Based on the above reasons, *θ_P_* would increase rapidly in the *p_ad_* range of 1% to 33% and decrease at 100% of *p_ad_* in comparison to *θ_P_* at 50, 67, and 83% of *p_ad_*.

This study proposed the LRC of photosynthesis when light is incident from both leaf surfaces. It would allow a detailed analysis of the effects of upward lighting in a PFAL and intra-canopy supplemental lighting in a greenhouse on Pn of plants.

### 4.3 Relationship between *p_dw_* and photosynthetic rate of a komatsuna canopy

From **Figure 9** and **Table 2**, under the conditions of 122 µmol s^−1^ of *Φ_lamps_*, when LAI was less than 2.5, the CPn was increased with more upward lighting (*p_dw_* = 20%). When LAI was greater than 3.5, the CPn was increased with more downward lighting than when LAI was less than 2.5 (*p_dw_* = 50, 100%). With greater than 244 µmol s^−1^ of *Φ_lamps_*, when LAI was less than 3.5, the CPn was increased with 50–70% of *p_dw_*. When LAI was greater than 4.5, the CPn was increased with more downward lighting (*p_dw_* = 80–100%). The possible causes could be *Φ_leaves_* and *SD_E_*.

CPn would be higher under the conditions of higher *Φ_lamps_* and lower *SD_E_*. When *Φ_lamps_* and *SD_E_* were high, there were locally high PPFD areas. In this case, the light-use efficiency of photosynthesis decreased in the high PPFD areas, and the CPn also decreased. Therefore, the *SD_E_* was considered to largely influence CPn under high *Φ_lamps_* conditions. This might reflect the fact that for higher *Φ_lamps_*, the CPn was likely to be the highest under the conditions with *p_dw_*, which resulted in a lower *SD_E_* (**Table 2** and **Figure 10B**).

Regarding the relationship between *p_dw_* and *Φ_leaves_*, in the simulation of this study, the lamps for upward lighting were placed between the plants, whereas the lamps for downward lighting were placed approximately 20 cm above the canopy. Therefore, the lamps for upward lighting were closer to the plants than those for downward lighting; therefore, it is considered that the light emitted from the lamps for upward lighting was less likely to leak out of the cultivation system and was more likely to be absorbed by the leaves in the canopy than the lamps for downward lighting. However, when the LAI was 4.5, the plant density was considerably high. Thus, when the LAI was 4.5, a larger percentage of light by upward lighting might be absorbed by the petioles instead of the leaf blades than when LAI was smaller than 4.5, resulting in lower *Φ_leaves_* (**Table S4**).

**Figure 12:**
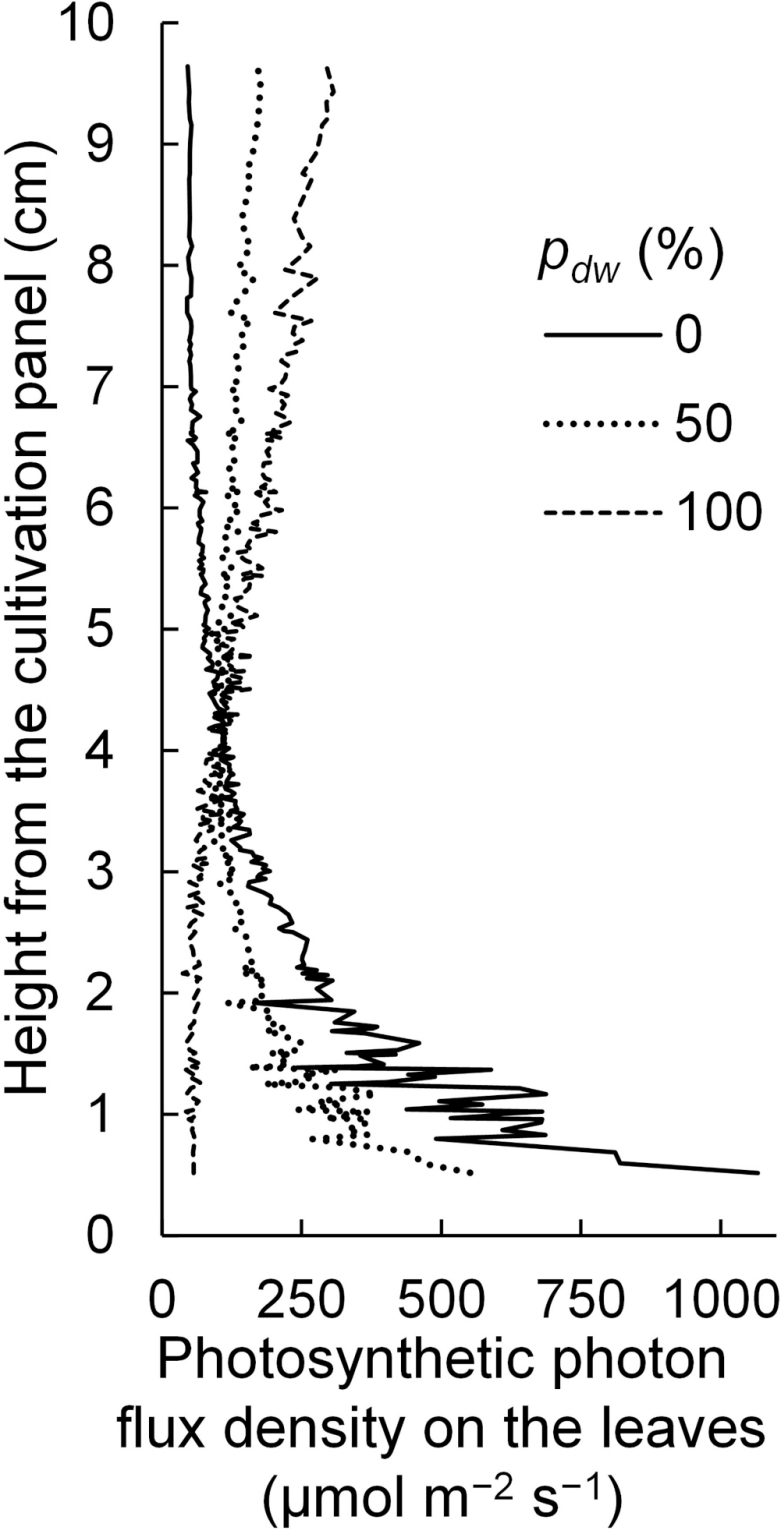
The vertical distribution of photosynthetic photon flux densities on the komatsuna leaves under different conditions, with the proportion (*p_dw_*) of the photosynthetic photon flux of lamps for downward lighting to that of whole lamps for downward and upward lighting (*Φ_lamps_*). The leaf area index and *Φ_lamps_* were 2.5 and 244 µmol s^−1^, respectively.

Regarding the *SD_E_*, it is considered that because the lamps for upward lighting were closer to the leaf surface, light tended to enter the leaves near the lamps. In this case, the PPFDs on the leaves near the lamps for upward lighting would be high, and the vertical PPFD gradient would be steeper than that in the case of downward lighting (**Figure 12**). In other words, under the simulation conditions in this study, the extinction coefficient of the canopy for upward lighting was larger than that for downward lighting.

As described above, we used optical simulations to estimate CPn under different *p_dw_* conditions and could provide effective *p_dw_* values to enhance CPn. The result shows that optical simulation is useful for evaluating the lighting design in a PFAL and analyzing the effects of the lighting design on photosynthesis. The plants used in this study were grown under downward light, whereas plants grown under upward light could be adapted to that light environment, and photosynthetic characteristics could be changed (Jiang et al., 2017; Saengtharatip et al., 2021; Yamori et al., 2021). Therefore, the parameters of LRC of the latter plants could be different from those of LRC of the plants in this study. Since the values of the parameters of LRC in the second and third leaves of 16 and 18 DAS were used to create interpolation formulas for the parameters of LRC, and the interpolation formulas were used to calculate the parameters of LRC in the other leaves on 18 DAS, it is unclear whether this interpolation method correctly estimates the parameters of the LRC for those leaves. In addition, since plant morphology can change with planting density, the shapes of 3D plant models would differ from actual plant shapes. Therefore, validation of the estimated CPn under various lighting conditions is necessary to improve the accuracy of the estimation.

### 4.4 Effect of upward lighting on the photosynthetic rate of a canopy

The results of this study showed that upward lighting might increase CPn. However, the gross photosynthetic rate of the canopy under conditions with a combination of downward and upward lighting was 1.08–1.13 times higher at most than under the conditions with downward lighting alone, even if the total PPFs of the lamps for upward and downward lighting are the same. If upward lighting is installed in a PFAL, the cost of lamps for upward lighting will increase. The depreciation accounted for 25–30% of the production cost in a PFAL (Kozai, 2019; Kozai and Niu, 2020), and the depreciation for lamps accounted for 30% of total depreciation (Uraisami, 2018). Therefore, the depreciation for lamps accounted for 7.5–9.0% of the production cost. On other hand, there was a linear relationship between Pn and the growth rate of the canopy (Hikosaka et al., 1999). If there was a proportional relationship between CPn and growth rate of the canopy, and the number of lamps for upward lighting was the same as that for downward lighting, enhancement of CPn by upward lighting would be valuable, even considering production cost (**Table S5**).

## 5 Conclusions

This study investigated the effects of total PPFD (*E*) on the adaxial and abaxial surfaces of a leaf, and the proportion (*p_ad_*) of PPFD on the adaxial surface to *E* on the Pn of a leaf. Pn varied with *p_ad_*. Under low *E* conditions, except when *E* was 30 or 70 µmol m^−2^ s^−1^, Pn increased as *p_ad_* increased owing to the difference in absorptances of the adaxial and abaxial surfaces of a leaf. Under high *E* conditions, Pn was maximized when *p_ad_* was 67–83% because of more photosynthetically efficient light distribution within the leaf. This would result from the difference in the PPFD gradient within a leaf when adaxial or abaxial surfaces are irradiated, as well as the photosynthetic capacity of chloroplasts in the adaxial or abaxial parts of a leaf. Next, we formulated an equation for the LRC of photosynthesis that took *p_ad_* into consideration.

Subsequently, using optical simulation and the formulated equation, we estimated CPn with downward and upward lighting. The simulations were performed under conditions with different LAI, total PPF (*Φ_lamps_*) of the whole lamps, and proportion (*p_dw_*) of the lamps’ PPF for downward lighting to *Φ_lamps_*. CPn increased by combining downward and upward lighting because the PPF (*Φ_leaves_*) of light incident on the canopy and the standard deviation (*SD_E_*) of PPFDs in the canopy improved. Under conditions with downward and upward lighting, CPn could be 1.08–1.13 times higher at most than that under the conditions with downward lighting alone. Because the depreciation of lamps for upward lighting accounts for 7.5–9.0% of the production cost in a PFAL, even if the depreciation of lamps for upward lighting increased, enhancement of CPn by upward lighting would be cost-effective.

In this study, we performed optical simulations under 220 conditions and evaluated them using CPn as an index. Moreover, we could provide effective *p_dw_* values to improve CPn and analyze the reason for this improvement. This shows that optical simulation is useful for evaluating the lighting design in a PFAL and analyzing the effects of the lighting design on the light environment and photosynthesis.

## Data availability statement

The original contributions presented in the study are included in the article/**Supplementary Material**. Further inquiries can be directed to the corresponding author.

## Author contributions

Conceptualization, methodology, design of the experiment, and funding acquisition: KS and EG. Performed the experiments and collected samples for analysis, parameter measurement, and statistical analysis of data: KS. Writing—original draft preparation: KS. Writing—review and editing: EG. Supervision: EG. Both authors contributed to the article and approved the submitted version.
