# Supplementary material for: Evaluation of the enhancement of photosynthetic rate in a komatsuna (Brassica rapa L. var. perviridis) canopy with upward lighting using an optical simulation in a plant factory with artificial light
Source: Front Plant Sci. 2023 Mar 24;14:1111338. doi: 10.3389/fpls.2023.1111338 (PMC10081495; doi:10.3389/fpls.2023.1111338)
Supplement: Supplementary file 1 [file DataSheet_1.docx]

Supplementary Material

# 1. Supplementary Figures


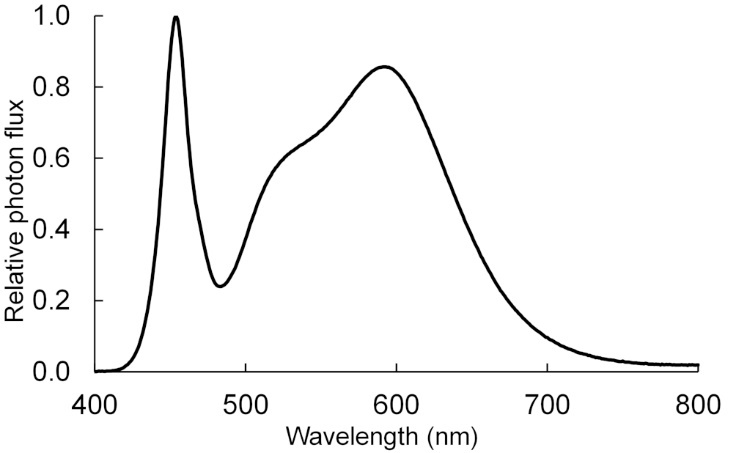


**Figure S1** The relative spectral photon distribution of a white light-emitting diode (XLX460NENT LE9, Panasonic Corporation, Osaka, Japan) measured by a spectroradiometer (USR-45, Ushio Inc., Tokyo, Japan). The maximum value was converted to 1.


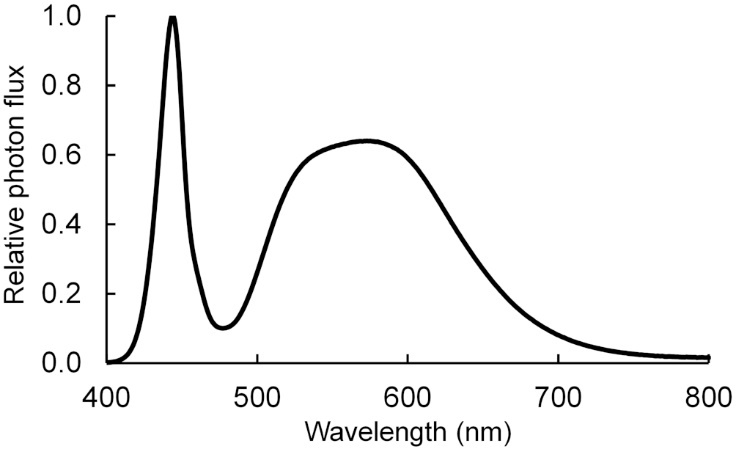


**Figure S2** The relative spectral photon distribution of a panel-type white light-emitting diode (ISLM-150X150-WW, CCS Inc., Kyoto, Japan) measured by a spectroradiometer (USR-45, Ushio Inc., Tokyo, Japan). The maximum value was converted to 1.

**2. Supplementary Tables**

**Table S1** List of symbols with descriptions.

| Symbol | Description | Unit |
| --- | --- | --- |
| *e* | Napier number | - |
| *E* | Total photosynthetic photon flux density on adaxial and abaxial surfaces of a leaf | μmol m^−2^ s^−1^ |
| *E_ab_* | Photosynthetic photon flux density on an abaxial surface of a leaf | μmol m^−2^ s^−1^ |
| *E_ad_* | Photosynthetic photon flux density on an adaxial surface of a leaf | μmol m^−2^ s^−1^ |
| *I_dw_(α)* | Photosynthetic photon intensity of light at *α* from a lamp for downward lighting | μmol s^−1^ sr^−1^ |
| *I_uw_(α)* | Photosynthetic photon intensity of light at *α* from a lamp for upward lighting | μmol s^−1^ sr^−1^ |
| *J* | Electron transport rate of a leaf | μmol m^−2^ s^−1^ |
| *p_ad_* | Proportion of the photosynthetic photon flux density on an adaxial surface of a leaf to total photosynthetic photon flux density on adaxial and abaxial surfaces of the leaf | % |
| *p_dw_* | Proportion of the photosynthetic photon flux of lamps for downward lighting to total photosynthetic photon flux of lamps for downward and upward lighting | % |
| *Pg(E)* | Gross photosynthetic rate of a leaf irradiated with light at *E* | μmol m^−2^ s^−1^ |
| *Pg(J)* | Gross photosynthetic rate of a leaf when electron transport rate was *J* | μmol m^−2^ s^−1^ |
| *P_max_* | Maximum gross photosynthetic rate of a leaf | μmol m^−2^ s^−1^ |
| *Pn(E)* | Net photosynthetic rate of a leaf irradiated with light at *E* | μmol m^−2^ s^−1^ |
| *Rd* | Dark respiration rate of a leaf | μmol m^−2^ s^−1^ |
| *SD_E_* | Standard deviation of *E* on leaves in a canopy | μmol m^−2^ s^−1^ |
| *α* | Vertical angle in a photometric curve | rad |
| *θ_P_* | Convexity of a light response curve of photosynthesis | - |
| *θ’_P_* | Standardized convexity of a light response curve of photosynthesis | - |
| *φ_P;ab_* | Initial slope of a light response curve of photosynthesis with 0% of *p_ad_* | - |
| *φ_P;ad_* | Initial slope of a light response curve of photosynthesis with 100% of *p_ad_* | - |
| *φ’_P_* | Standardized initial slope of a light response curve of photosynthesis | - |
| *φ’_P;ab_* | Standardized initial slope of a light response curve of photosynthesis with 0% of *p_ad_* | - |
| *φ’_P;ab_* | Standardized initial slope of a light response curve of photosynthesis with 100% of *p_ad_* | - |

**Table S2** The predetermined values of the environmental conditions during cultivations.

| Environment element | Predetermined value | |
| --- | --- | --- |
|  | 0–7  days after sowing | 7–18  days after sowing |
| Light period (h) | 16 | 16 |
| Photosynthetic photon flux density (µmol m^−2^ s^−1^) | 200 | 250 |
| Air temperature (°C, Light/Dark) | 25/20 | 25/20 |
| Relative humidity (%) | 73 | 73 |
| CO_2_ concentration (µmol mol^−1^) | 1000 | 1000 |

**Table S3** The error indices of the calculated net photosynthetic rates with the method described in Section **2.3.2**.

|  | MAE  (µmol m^−2^ s^−1^) | MAPE (%) | RMSE  (µmol m^−2^ s^−1^) | R^2^ |
| --- | --- | --- | --- | --- |
| 16 DAS-2nd | 1.24 | 6.61 | 1.71 | 0.9869 |
| 16 DAS-3rd | 0.81 | 4.43 | 1.05 | 0.9951 |
| 18 DAS-2nd | 1.25 | 7.59 | 1.70 | 0.9852 |
| 18 DAS-3rd | 0.71 | 3.75 | 1.12 | 0.9949 |

MAE, MAPE, RMSE, and R^2^ indicate the mean absolute error, mean absolute percentage error, root mean squared error, and adjusted coefficient of determination (*n* = 149–184), respectively. A total of 149–184 values of measured photosynthetic rates were used to calculate the error indices each day after sowing (DAS) and leaf position (2nd and 3rd). The values of net photosynthetic rates calculated using the method described in Section **2.3.2** were used to calculate the error indices.

**Table S4** The values of proportion of the photosynthetic photon flux incident on the petioles to the total photosynthetic photon flux incident on the leaves and petioles at each leaf area index (LAI) condition.

|  |  | *p_dw_* (%) | |
| --- | --- | --- | --- |
|  |  | 0 | 100 |
| LAI | 0.5 | 37 | 9.4 |
|  | 1.5 | 34 | 8.3 |
|  | 2.5 | 34 | 6.3 |
|  | 3.5 | 27 | 3.3 |
|  | 4.5 | 41 | 1.2 |

**Table S5** The cost-effectiveness of upward lighting.

| *Φ_lamps_* (µmol s^−1^) | LAI_max_ | *p_dw;max_* (%) | *CPn_max_*/*CPn_dw_* | *D_uw+dw_*/*D_dw_* |
| --- | --- | --- | --- | --- |
| 122 | 0.5 | 20 | 1.28 | 4.12–4.74 |
| 244 | 1.5 | 50 | 1.12 | 2.33–2.60 |
| 366 | 2.5 | 60 | 1.10 | 2.07–2.28 |
| 488 | 2.5 | 70 | 1.09 | 2.05–2.26 |

*p_dw_* indicates the proportion of the photosynthetic photon flux of lamps for downward lighting to that of whole lamps for downward and upward lighting (*Φ_lamps_*). LAI_max_ and *p_dw;max_* indicate the values of leaf area index and *p_dw_* where the net photosynthetic rate of a canopy (CPn) was maximized in each *Φ_lamps_* condition. *CPn_max_* and *CPn_dw_* indicate the values of CPn when *p_dw_* was *p_dw;max_* and 100%, respectively. The values of *D_uw+dw_*/*D_dw_* mean maximum increment rate in depreciation for lamps by upward lighting and were calculated as follows.

$$\frac{D_{uw+dw}}{D_{dw}}=1+\frac{\frac{{CPn}_{max}}{{CPn}_{dw}}-1}{\frac{p_{D;dw}}{100}}$$

*D_uw+dw_* and *D_dw_* were the depreciations of lamps for upward and downward lighting and that of lamps for downward lighting, and *p_D;dw_* (%) was the proportion of depreciation of lamps for downward lighting to the total production cost in a plant factory with artificial light. In this study, *p_D;dw_* was assumed as 7.5–9.0%. If the number of lamps for upward lighting is the same as that for downward lighting, *D_uw+dw_*/*D_dw_* is 2.
